# Ultrasound-Guided Endoluminal Thermoablation of the Frontal Branch of the Superficial Temporal Artery Using a 1470-nm Laser: A Case Report

**DOI:** 10.7759/cureus.108371

**Published:** 2026-05-06

**Authors:** Marcelo F Lima, Mariah P Rios Lima

**Affiliations:** 1 Phlebology, Clínica Dr. Marcelo Lima - Medicina Vascular, Manaus, BRA

**Keywords:** endovascular laser, facial aesthetics, laser thermoablation, superficial temporal artery, ultrasound-guided procedure

## Abstract

In modern life, the demand for aesthetic procedures is growing, driven by the exposure of one’s image on social media and the desire for facial harmony that is as close as possible to a social ideal. Dilated veins on the face can cause significant aesthetic discomfort. The 1064 nm transdermal laser is effective at eliminating telangiectasias and reticular veins measuring 2-3 mm, although reticular veins may require several sessions for satisfactory results. For larger veins, especially in the forehead region, thermoablation with a 1470-nm laser is recognized for its safety and efficiency. This study reports a case of aesthetic discomfort caused by the frontal branches of the superficial temporal artery in a 72-year-old woman, treated using principles similar to those used for frontal veins. The procedure provided an excellent aesthetic outcome, representing, to the best of our knowledge, the first reported use of this technique for this purpose.

## Introduction

Prominent facial vessels are a frequent aesthetic concern in clinical practice, particularly in aging patients in whom progressive loss of subcutaneous fat increases vascular visibility. While telangiectasias and small reticular veins can be effectively treated with transdermal laser techniques, larger-caliber vessels in the frontal region remain more challenging to manage. In addition, prominent arterial structures, such as the frontal branches of the superficial temporal artery, represent a less commonly addressed but clinically relevant source of aesthetic discomfort, with limited treatment options described in the literature [1-3].

In this context, the face stands out for being constantly exposed, not only on social networks but also in real life. More visible veins in this region cause profound aesthetic discomfort for many people, especially women, as they give the appearance of premature aging in individuals still considered young, and the complaint worsens in older people, in whom the decrease in facial fat accentuates the visibility of these veins. Complaints of aesthetic discomfort due to prominent facial veins are common in vascular surgery clinics, especially those focused on venous treatments with aesthetic goals. Lasers allow us to treat everything from telangiectasias to reticular veins between 2 and 3 mm in diameter with a 1064-nm Nd:YAG laser, with less satisfactory results as vein caliber increases. Larger veins, such as frontal veins, are treated safely and effectively with the 1470-nm diode laser [4].

In older patients, the loss of facial fat further highlights the prominence of these veins. In many individuals, branches of the superficial temporal arteries (STA) are also very prominent, causing significant aesthetic discomfort. In these cases, the technical alternatives described in the literature are filling the area surrounding the vessels with biostimulating dermal fillers or surgical ligation with or without extraction of the arterial branches [5-8].

Here, we report the use of a 1470-nm diode laser to treat the frontal branches of the superficial temporal artery (FBSTA) in a patient who experienced significant aesthetic discomfort due to the prominent appearance of these vessels. The 1470-nm diode laser is particularly suitable for the treatment of larger or more prominent vessels due to its high absorption by water, which is abundant in both blood and vessel wall. This wavelength enables efficient and controlled thermal injury of the endothelium, resulting in reliable vessel occlusion. In addition, when delivered through endoluminal fibers, especially radial fibers, it enables uniform energy distribution along the vessel circumference, minimizing the risk of perforation and reducing damage to surrounding tissues. These properties make the 1470-nm laser especially effective and safe for treating vessels with larger diameters compared to transdermal approaches. Notably, interventions involving arterial structures carry a higher risk profile than venous procedures, requiring precise anatomical knowledge and careful vascular assessment.

Written informed consent for publication, including clinical data and images, was obtained from the patient, and the signed consent form has been submitted as supplementary material for editorial review. In accordance with institutional policies, formal ethical committee approval was not required for this single case report.

## Case presentation

**Figure 1:**
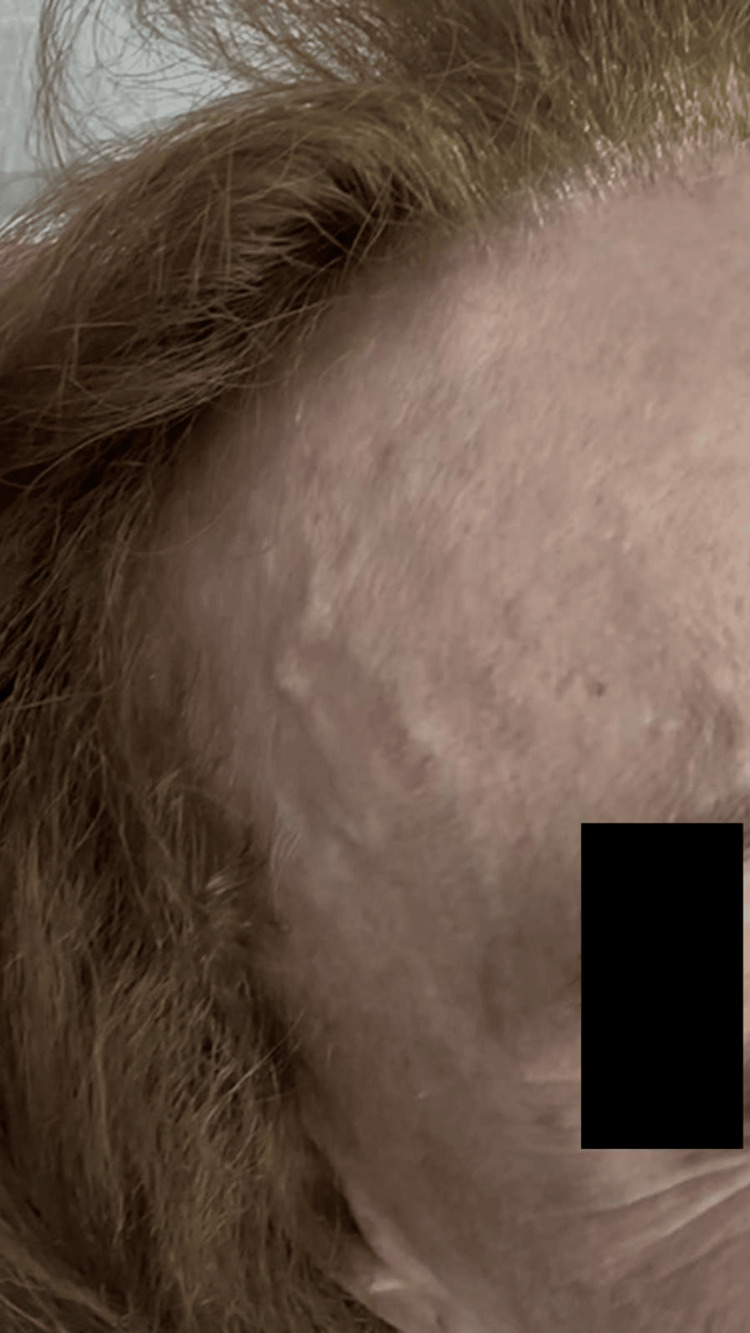
Preprocedural clinical appearance showing prominent, visibly pulsatile frontal branch of the right superficial temporal artery, responsible for the patient’s aesthetic concern.

**Figure 2:**
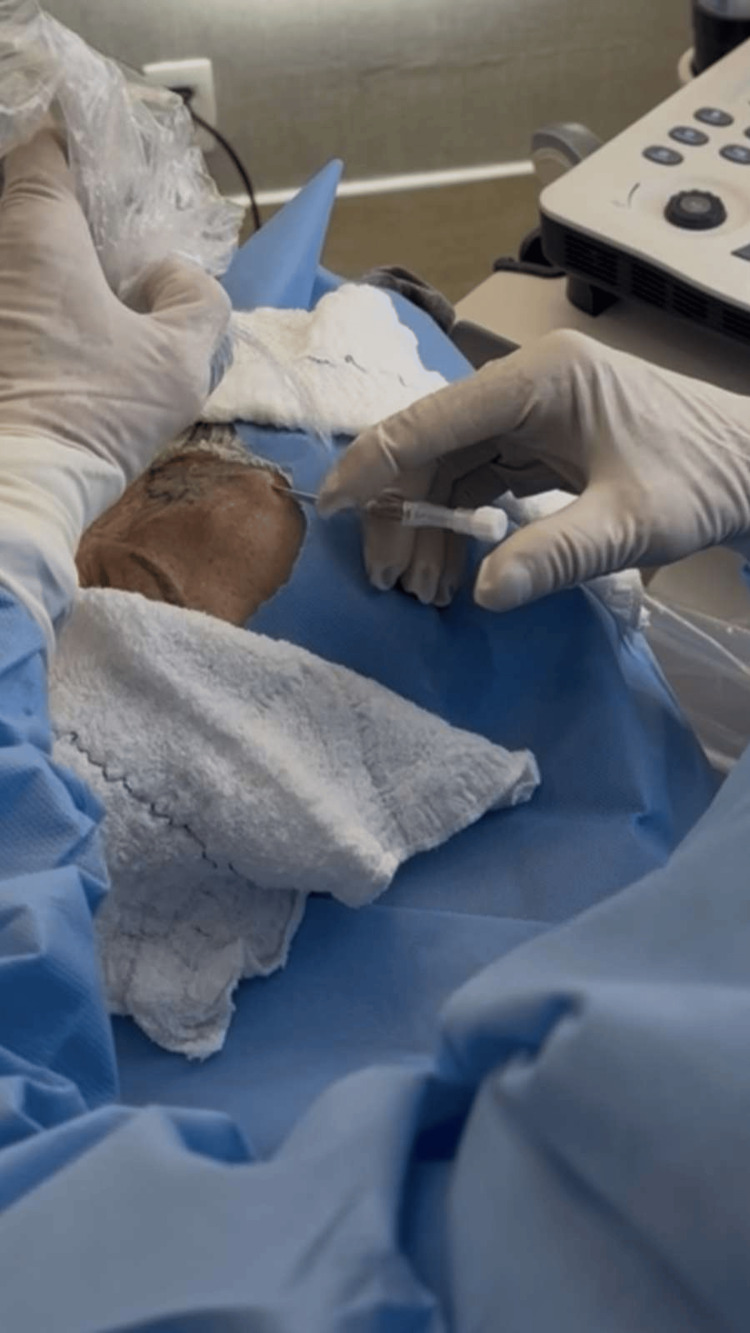
The frontal branch of the superficial temporal artery was cannulated, and a 400 µm radial fiber was advanced into the arterial lumen.

In March, 2025, a 72-year-old woman presented to our clinic complaining of significant aesthetic discomfort caused, in her perception, by dilated veins on her face. On physical examination, we found that these were, in fact, frontal branches of both STA, a diagnosis confirmed by Doppler ultrasound, which showed arteries with a maximum diameter of 1.5 mm, most measuring around 1 mm (Figures 1, 2). For aesthetic purposes, we initially recommended filling the temporal region with biostimulators or surgical ligation of the branches, but both options were declined by the patient.

We discussed with the patient the possibility of treating these vessels with intra-arterial thermoablation using a 1470-nm laser, similar to the treatment of frontal veins, emphasizing the novel nature of this technique in this situation, as well as its possible complications, such as hematomas and bleeding that may require surgical intervention, since to the best of our knowledge, this technique had not previously been employed for these situations. The patient opted to undergo the treatment, fully aware of the potential benefits and risks as discussed.

**Figure 3:**
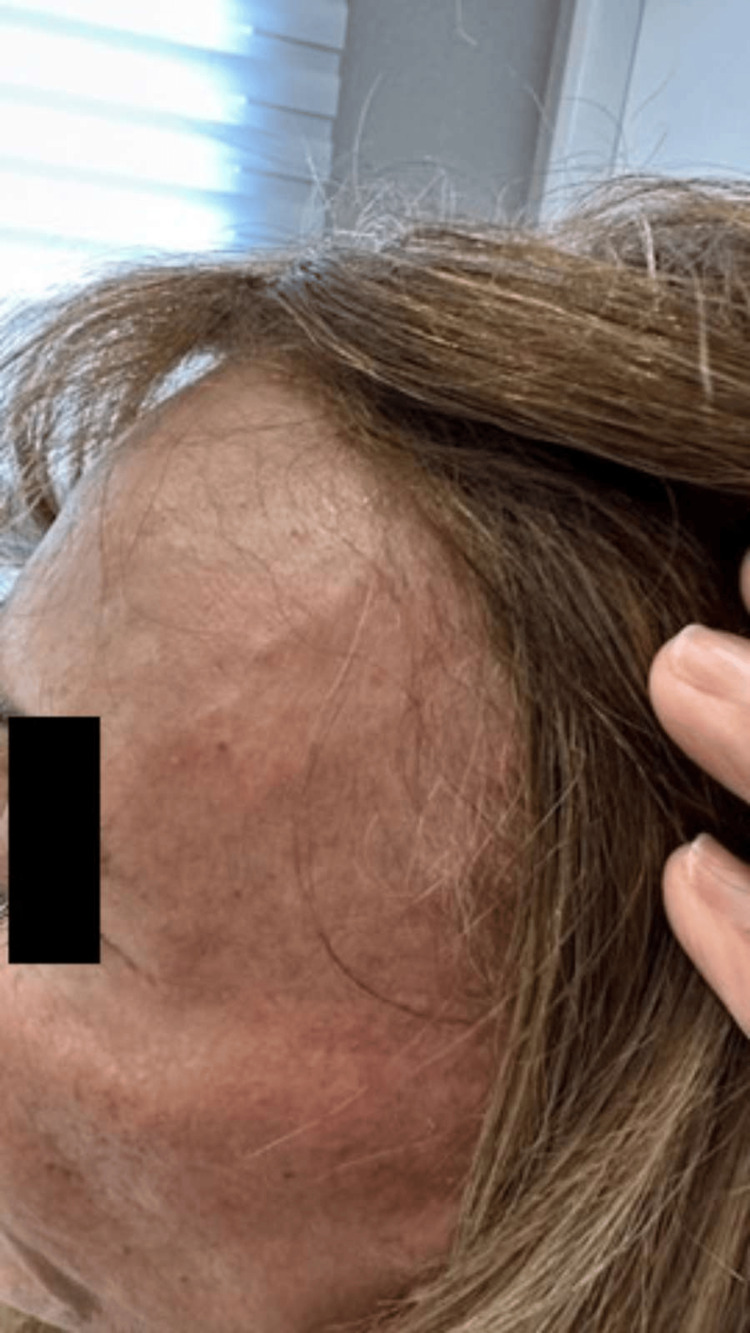
Preprocedural clinical appearance of the left side, demonstrating a prominent and visibly pulsatile frontal branch of the superficial temporal artery, similar to the contralateral side.

**Figure 4:**
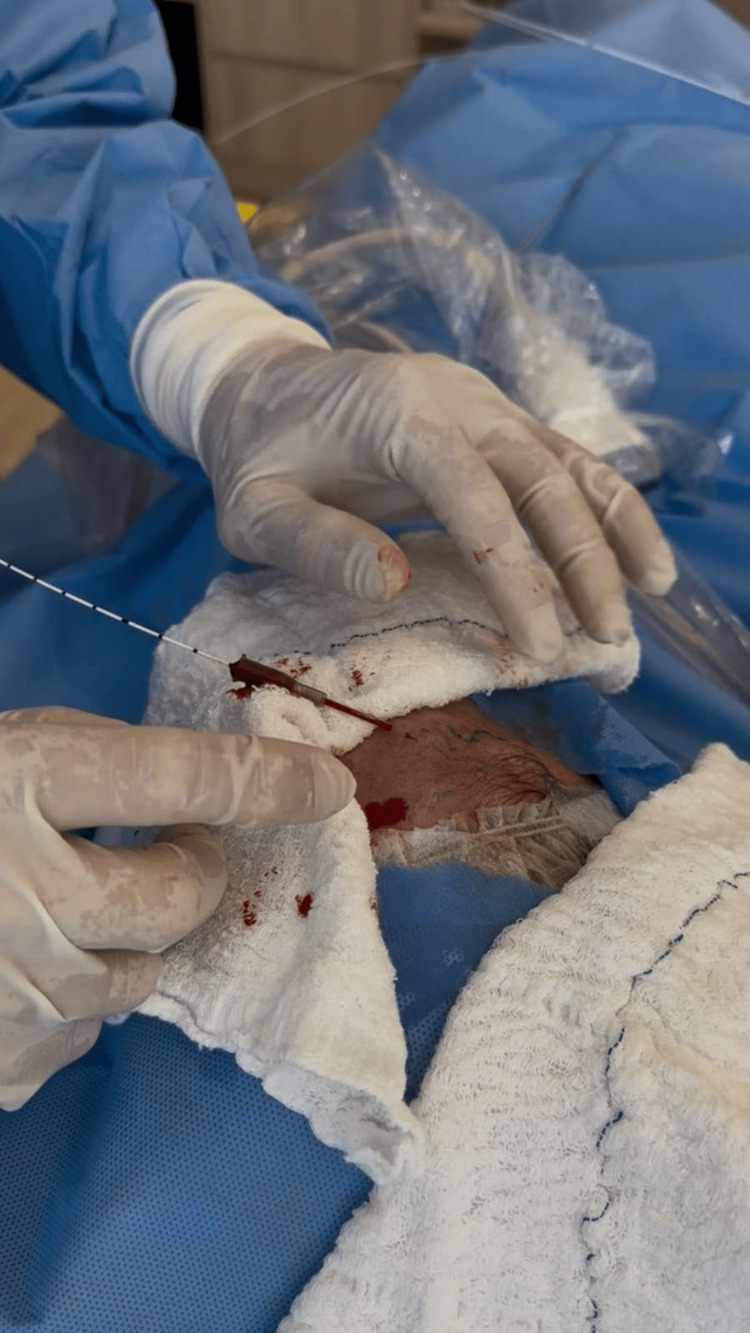
Cannulation of the frontal branch of the superficial temporal artery with advancement of a 400 µm radial fiber within the arterial lumen under ultrasound guidance.

**Figure 5:**
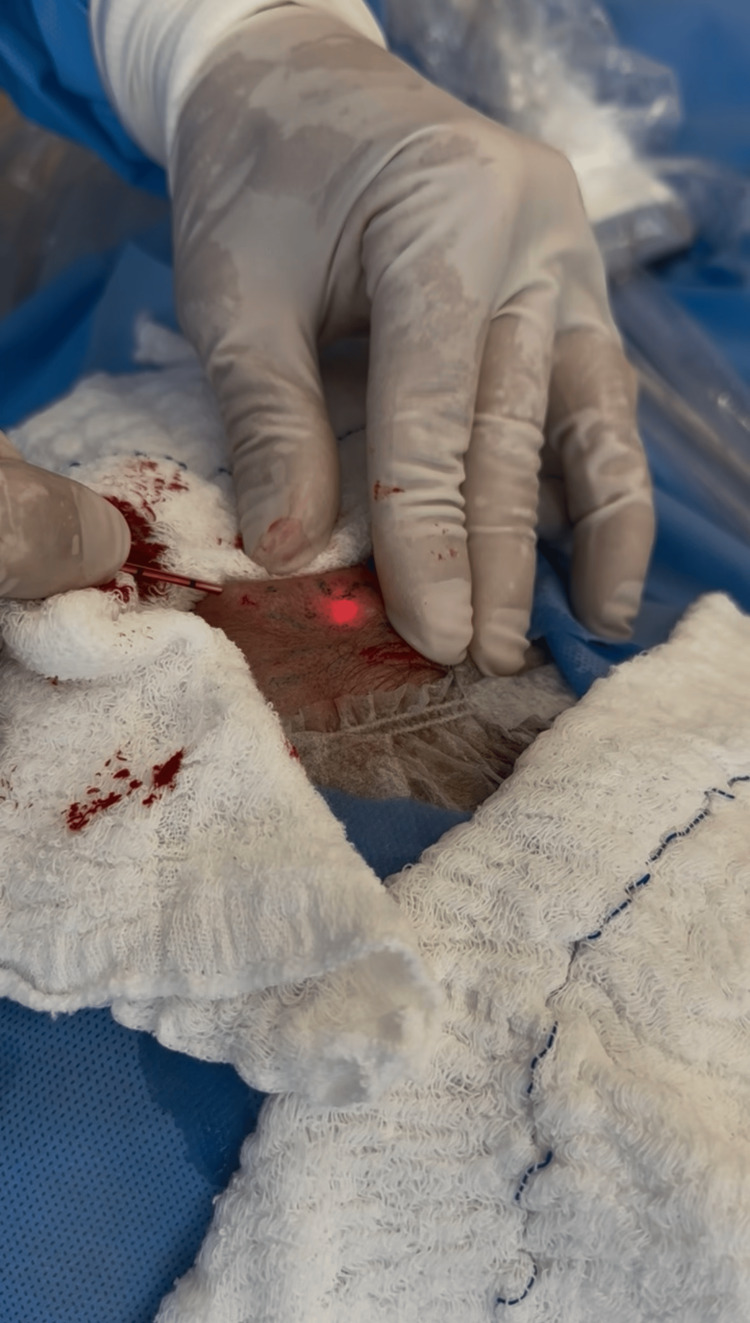
Endolaser thermoablation of the frontal branch of the superficial temporal artery.

The treatment was performed with ultrasound-guided punctures of the arterial branches using a 16-gauge peripheral intra-arterial catheter and thermoablation of the segments with a 400 µm slim radial fiber and 4 J energy, with an average linear endovenous energy density (LEED) of 30 J/cm, under tumescent anesthesia (Figures 3-5). There was no hematoma or bleeding during or immediately after the procedure. Absence of flow in the treated segments was confirmed by Doppler ultrasound. The patient was referred for dermatologic physiotherapy the day after the procedure, during which she underwent three sessions of lymphatic drainage, with a scheduled reevaluation.

**Figure 6:**
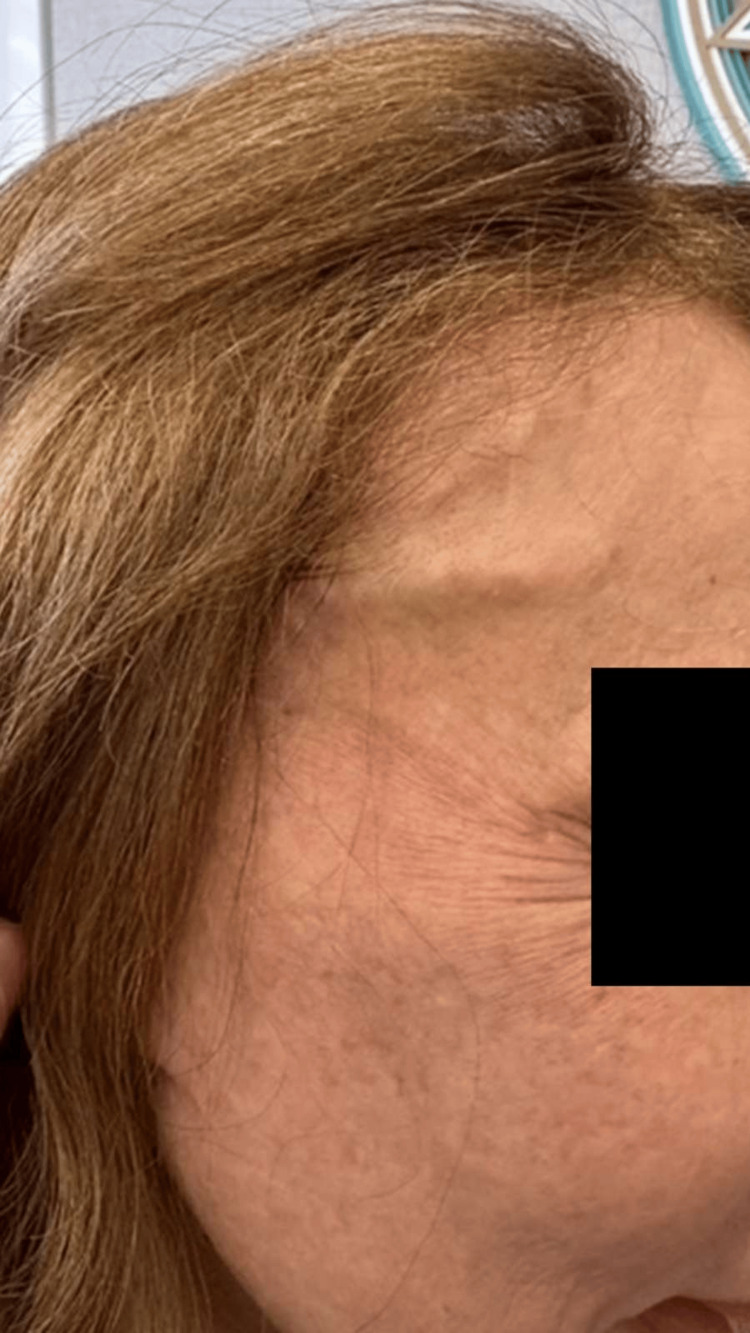
Early postprocedural clinical appearance seven days after endoluminal laser thermoablation, presenting edema along the course of the FBSTA on each side without hematoma or ecchymosis. FBSTA: frontal branch of superficial temporal artery

**Figure 7:**
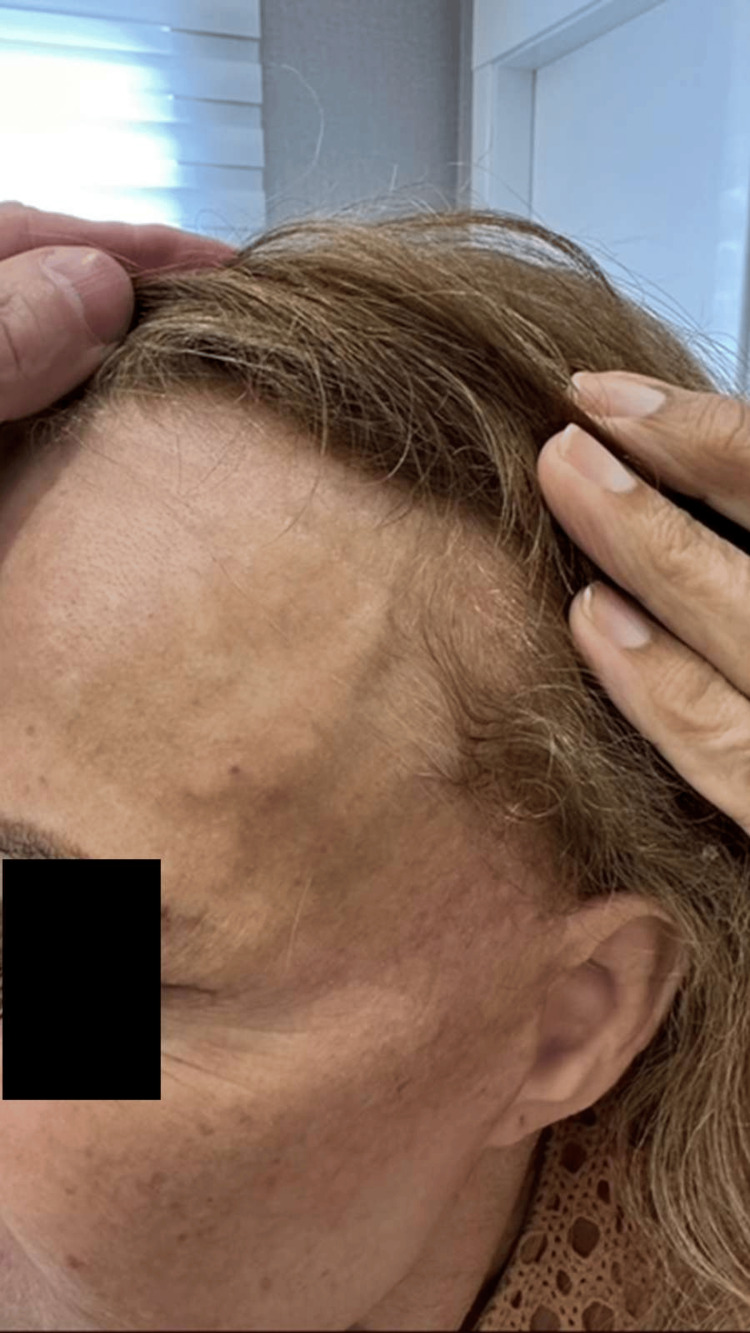
Early postprocedural appearance (seven days) on the left side, demonstrating similar edema along the treated arterial pathway, with preserved skin integrity and no visible complications.

**Figure 8:**
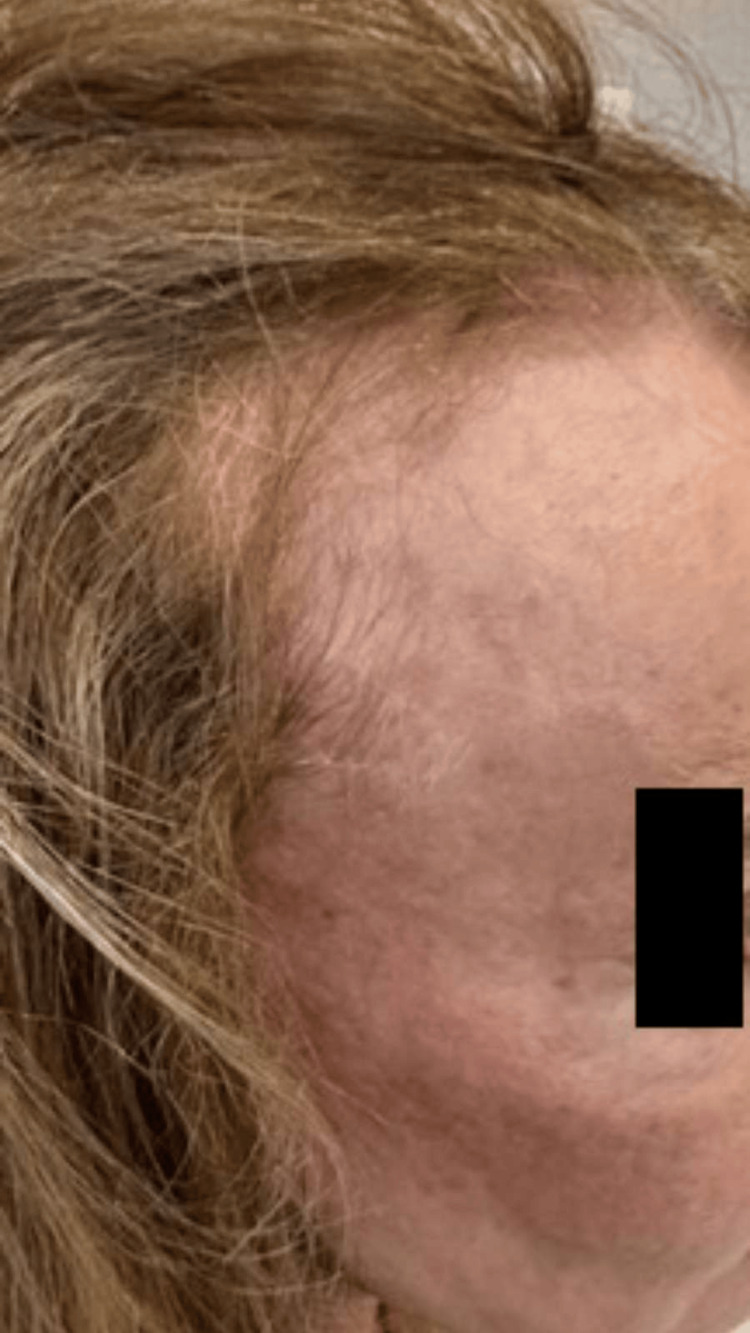
Late clinical result seven months after the procedure, demonstrating complete resolution of the previously visible arterial branches, an excellent aesthetic outcome, and sustained cosmetic improvement with high patient satisfaction.

**Figure 9:**
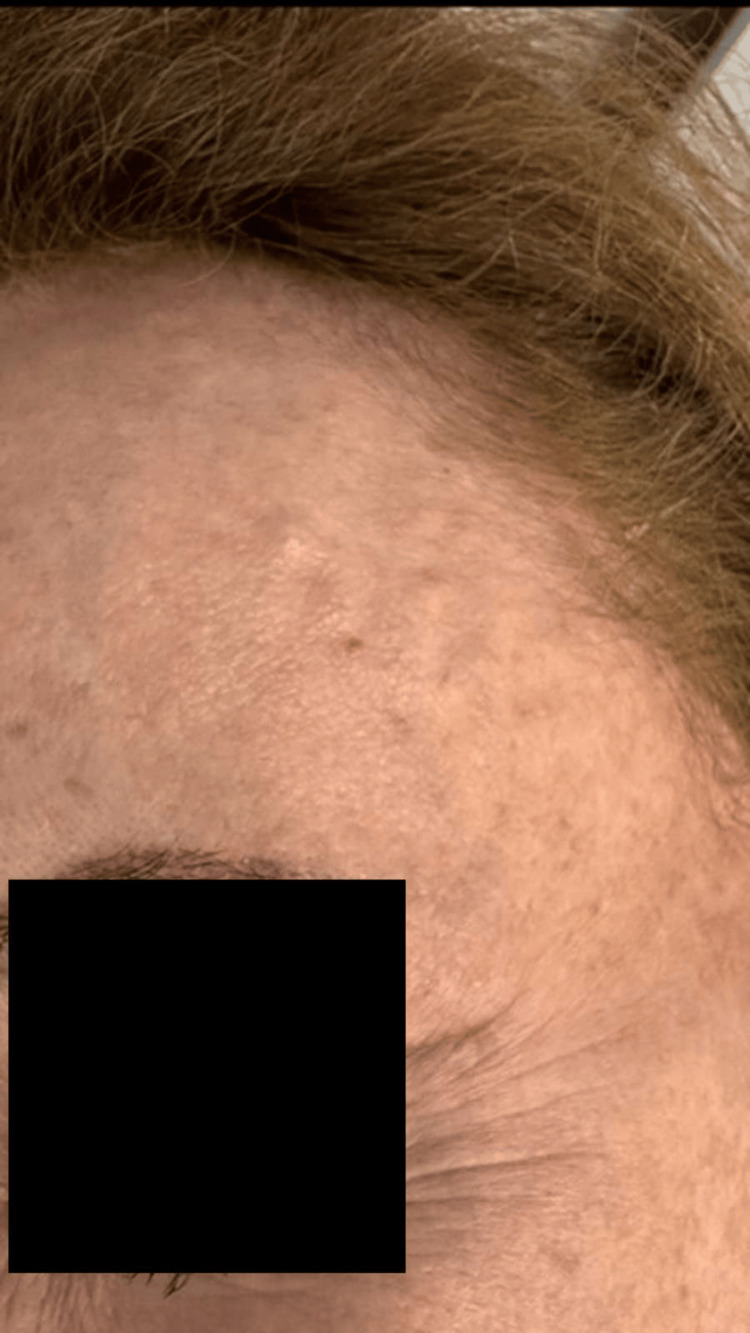
Late postprocedural result (seven months) on the left side, confirming sustained occlusion of the treated vessel and marked aesthetic improvement consistent with the contralateral side.

The patient returned for evaluation eight days after the procedure, presenting with edema along the course of the FBSTA on each side, but with no pain, bruising, or detectable arterial flow on Doppler ultrasound (Figures 6, 7). She was advised to continue physiotherapy for the regression of the edema. Due to professional commitments, the patient left the city and returned seven months after the treatment, presenting with an excellent aesthetic result and a high degree of satisfaction (Figures 8, 9).

## Discussion

The technique of thermoablation of unsightly frontal veins is well established, showing efficiency and safety when performed by professionals with experience in guided punctures and management of an endovenous laser [4]. However, aesthetic concern due to the prominence of arterial branches on the forehead leaves limited therapeutic options for achieving satisfactory aesthetic outcomes, with the remaining alternatives being vessel ligation-with or without excision of the branches-or volume restoration using biostimulatory fillers to mask the vessels [5-8].

Understanding the facial vascular anatomy is essential for aesthetic interventions in areas of concern, particularly in the use of dermal fillers or autologous fat grafting [1,9]. The temporal region exhibits intricate vascular anatomy because numerous vessels course through its various layers, and there are also strong arterial and venous connections to the internal carotid artery and the cavernous sinus. The superficial temporal artery is responsible for the vascular supply of extensive portions of the scalp and face. Unintentional vessel damage may lead to hemorrhage or to the progressive formation of a pseudoaneurysm, which has the potential to rupture, causing severe bleeding [2].

The superficial temporal artery (STA), a terminal branch arising from the external carotid artery, supplies the parietal and temporal regions. It ascends in the interval between the tragus and the posterior root of the zygomatic arch, where it bifurcates into two principal branches: the posterior parietal branch and the anterior frontal branch (AFB). The AFB provides blood to the anterior portion of the temporal muscle, located posterior to the superior temporal area [10]. The course of the STA trunk maintains a close anatomical relationship with the auriculotemporal nerve, which is a branch of the mandibular nerve [1-3,9-11].

The AFB is regarded as the most significant artery for any designed clinical procedures in this temporal region, in which numerous studies have been reported, including studies involving the morphological description of this vascular area [9].

Water within blood and a vessel wall serves as the principal chromophore for the 1470-nm laser, enabling the induction of irreversible thermal damage and subsequent occlusion of the targeted vessels. Even distribution of laser energy along the vessel’s inner surface is essential during treatment and can be optimized by employing specialized lenses at the fiber tip (radial fibers). Numerous studies have demonstrated that thermoablation using radial fibers significantly reduces the rate of postoperative complications, while also resulting in reduced postoperative pain and faster recovery when compared with procedures performed with bare-tip fibers [12].

To ensure the safety of procedures performed in the temporal region, an in-depth knowledge of the area’s specific vascular anatomy and awareness of high-risk zones for vascular and nerve complications, along with the use of ultrasonography to increase safety during procedures, should be concerns for any professional intending to work in this area. In the upper lateral forehead, beyond the main vascular bifurcation and as the artery becomes more superficial within the subcutaneous plane, spatial separation from the deeper nerve branches may increase. This creates a potential “selective safe window,” particularly when confirmed by ultrasound. Nevertheless, even in this region, anatomical variability must be acknowledged and appreciated. Studies have demonstrated that the temporal branch may exhibit accessory arteries or more superficial trajectories in a minority of individuals, reinforcing the necessity of individualized mapping rather than reliance on textbook representations [1,13].

The development of a pseudoaneurysm or an arteriovenous fistula involving the superficial temporal vessels, as well as bleeding, represented the most frequent complications following surgical procedures, particularly after rhytidectomy and malarplasty. In contrast, vascular complications affecting the cerebral and ophthalmic arteries were more commonly associated with the use of autologous fat grafting and filler injections. Reports have also described pseudoaneurysm formation after partial arterial transection or needle puncture during local anesthetic infiltration. Cases of STA pseudoaneurysm have been documented following both rhytidectomy and thread lifting. Arteriovenous fistulas have likewise been reported after surgical interventions, including rhytidectomy, reduction malarplasty, and cartilage graft removal during rhinoplasty. The susceptibility of the STA to injury may be attributed to the lack of protective tissue separating it from the underlying bone [2].

Endovascular laser thermoablation has well-known complications in the venous territory, such as perforations of treated vessels with bruising and hematomas, and thermal injuries to peripheral nerves due to the proximity to the treated vessels. However, when the artery is confined intraluminally, surrounded by tumescent solution, and treated using controlled radial energy dispersion, lateral thermal spread is limited. Thus, procedural safety is maximized when the following three protective layers are respected: anatomical mapping, ultrasound guidance, and perivascular tumescence [14].

The anatomical relationship between the STA, AFB, and facial nerves is well established. As the most superior terminal branch of the external carotid artery, the STA emerges from deeper planes in the superficial temporal fascia in all observed cases, approximately 1 cm anterior and 1 cm superior to the tragus apex. At this location, its course shows a close association with the auriculotemporal nerve, which originates from the mandibular nerve [3,11].

Understanding the spatial relationship between the AFB and the temporal branch of the facial nerve (TBFN) is essential for minimizing complications during AFB puncture. Certain authors have proposed using a line drawn from the earlobe to the lateral extremity of the eyebrow to approximate the trajectory of either the AFB or the TBFN. In the temporal region, the TBFN is generally positioned anterior and inferior relative to the AFB [15,16].

Typically, the TBFN lies about 1-2 cm inferior and anterior to the AFB within the temporal region. Concerning fascial layer relationships, the STA courses through the superficial temporal fascia, whereas the TBFN travels along the undersurface of this fascial layer (fibroadipose plane). However, punctures performed excessively close to the lateral orbital margin increase the risk of postoperative dysfunction of the facial nerve. A defined risk zone can be established by tracing an oblique line through midpoints between the AFB and TBFN, located roughly 6.0 and 4.5 cm posterior to a vertical line drawn through the lateral orbital margin at the levels of the lateral canthus and the supraorbital margin, respectively [17].

## Conclusions

Although this is a report of a single case, thermoablation of the frontal branches of the STA using a 1470-nm laser for aesthetic purposes proved technically feasible and yielded an excellent cosmetic result. The similarity of the procedure to the treatment of frontal veins suggests potential reproducibility and may represent an additional option in facial aesthetics; however, further studies with larger samples are needed to confirm its safety and clinical applicability. We emphasize, however, that the practitioner must not only master the vascular and neural anatomy of the frontotemporal region but also have technical excellence in ultrasound-guided punctures and endovascular laser management, as inadvertent perforations of arterial branches can lead to bleeding and hematomas that may require surgical intervention to control hemorrhage, with evident aesthetic damage to the patient.

## References

[1] Kliniec K, Domagała Z, Kempisty B, Szepietowski JC (2024). Arterial vascularization of the forehead in aesthetic dermatology procedures: a review. J Clin Med.

[2] Daskalopoulou D, Matsas A, Chrysikos D, Troupis T (2022). The superficial temporal artery: anatomy and clinical significance in the era of facial surgery and aesthetic medicine. Acta Med Acad.

[3] Cotofana S, Lachman N (2019). Arteries of the face and their relevance for minimally invasive facial procedures: an anatomical review. Plast Reconstr Surg.

[4] Pereira CE, Rover CA, Whiteley MS (2021). Endovenous thermal ablation of prominent central forehead veins (supratrochlear veins). Dermatol Surg.

[5] Juhász ML, Marmur ES (2015). Temporal fossa defects: techniques for injecting hyaluronic acid filler and complications after hyaluronic acid filler injection. J Cosmet Dermatol.

[6] Moradi A, Shirazi A, Perez V (2011). A guide to temporal fossa augmentation with small gel particle hyaluronic acid dermal filler. J Drugs Dermatol.

[7] Nikolis A, Enright KM, Nguyen Q, Cotofana S (2023). The suitability of a large particle hyaluronic acid filler for the treatment of temporal hollowing. Dermatol Surg.

[8] Whiteley MS (2021). A double-ligation technique to remove prominent frontal branches of the superficial temporal artery. Dermatol Surg.

[9] Park HJ, Lee JH, Jung W (2022). The superficial temporal artery and zygomatico-orbital artery: superficial arterial distribution of the anterior temple area. Biomed Res Int.

[10] Lee JG, Yang HM, Hu KS, Lee YI, Lee HJ, Choi YJ, Kim HJ (2015). Frontal branch of the superficial temporal artery: anatomical study and clinical implications regarding injectable treatments. Surg Radiol Anat.

[11] Fagni N, Valli L, Nittari G (2025). Superficial temporal artery: anatomical variation and its clinical significance. J Vasc Dis.

[12] Artemov SA, Belyaev AN, Bushukina OS (2022). Morphological changes of veins and perivenous tissues during endovenous laser coagulation using 2-μm laser radiation and various types of optical fibers. J Vasc Surg Venous Lymphat Disord.

[13] Tansatit T, Phumyoo T, Jitaree B (2018). Ultrasound evaluation of arterial anastomosis of the forehead. J Cosmet Dermatol.

[14] Viarengo G, Martins GM, Viarengo LM (2024). Complicações do laser endovenoso. Laser Em Angiologia E Cirurgia Vascular.

[15] Albertini JG, Ramsey ML, Marks VJ (1999). Temporal artery biopsy in a dermatologic surgery practice. Dermatol Surg.

[16] Gunawardene AR, Chant H (2014). Facial nerve injury during temporal artery biopsy. Ann R Coll Surg Engl.

[17] Shin KJ, Shin HJ, Lee SH, Koh KS, Song WC (2018). Surgical anatomy of the superficial temporal artery to prevent facial nerve injury during arterial biopsy. Clin Anat.

